# The Zoonotic Potential of Chronic Wasting Disease—A Review

**DOI:** 10.3390/foods12040824

**Published:** 2023-02-15

**Authors:** Michael A. Tranulis, Morten Tryland

**Affiliations:** 1 Department of Preclinical Sciences and Pathology, Faculty of Veterinary Medicine, Norwegian University of Life Sciences, 5003 As, Norway; 2 Department of Forestry and Wildlife Management, Faculty of Applied Ecology, Agricultural Sciences and Biotechnology, Inland Norway University of Applied Sciences, 2480 Koppang, Norway

**Keywords:** cervids, CWD, wildlife, zoonosis

## Abstract

Prion diseases are transmissible neurodegenerative disorders that affect humans and ruminant species consumed by humans. Ruminant prion diseases include bovine spongiform encephalopathy (BSE) in cattle, scrapie in sheep and goats and chronic wasting disease (CWD) in cervids. In 1996, prions causing BSE were identified as the cause of a new prion disease in humans; variant Creutzfeldt-Jakob disease (vCJD). This sparked a food safety crisis and unprecedented protective measures to reduce human exposure to livestock prions. CWD continues to spread in North America, and now affects free-ranging and/or farmed cervids in 30 US states and four Canadian provinces. The recent discovery in Europe of previously unrecognized CWD strains has further heightened concerns about CWD as a food pathogen. The escalating CWD prevalence in enzootic areas and its appearance in a new species (reindeer) and new geographical locations, increase human exposure and the risk of CWD strain adaptation to humans. No cases of human prion disease caused by CWD have been recorded, and most experimental data suggest that the zoonotic risk of CWD is very low. However, the understanding of these diseases is still incomplete (e.g., origin, transmission properties and ecology), suggesting that precautionary measures should be implemented to minimize human exposure.

## 1. Introduction

Zoonoses are human diseases caused by pathogens derived from natural vertebrate animal reservoirs either directly or via intermediate animal hosts. It is estimated that of the emerging infectious diseases in humans after 1940, at least 60% are zoonotic and that the majority of these (>70%) are caused by pathogens originating in wildlife [1].

Prions are unique pathogens consisting of protein aggregates that cause incurable transmissible neurodegenerative diseases in humans and some other mammalian species [2]. These diseases (Table 1 and Table 2) are, with three notable exceptions, very rare and, although transmissible, not normally contagious. Rather, they occur naturally as sporadic and/or genetic diseases, although outbreaks can occur under conditions created by humans (e.g., recycling of prion infected feedstuff or iatrogenic) [3]. The exceptions are classical scrapie in sheep, chronic wasting disease (CWD) in deer, and camelid prion disease in dromedary camels [4]. For these diseases, the infectious prions are present at high titers in lymphoid organs [5,6,7] and detectable in bodily excretions, allowing horizontal (nose-to-nose) or indirect transmission via contaminated environs [8]. These prion diseases, therefore, pose particular problems, not only because infectious prions are abundantly present in musculature and other edible tissues, thus entering the human food chain, but also because the release of prions to the environment is building a transmission potential over time, contributing to increased infection pressure for animals sharing these habitats [9,10,11]. The latter problem is compounded by the extraordinary physiochemical stability of prions, making prion-contaminated environs a long-term challenge [9,12,13].

Natural transmission of CWD occurs most frequently between genetically susceptible individuals of the same or a closely related species [33]. This is explained by the molecular composition of prions and their peculiar way of propagation [34]. The normal cellular prion protein (PrP^C^), encoded by the *PRNP* gene [35,36], is a cell surface protein expressed in most tissues and at high levels in the central and peripheral nervous systems [37]. Its physiological functions are not fully understood [38,39,40]. Prions are multi-molecular aggregates of a misfolded conformer (termed PrP^Sc^) of PrP^C^ [41,42]. In prion propagation, PrP^Sc^ binds to PrP^C^ and templates the misfolding of PrP^C^ into the PrP^Sc^ conformational state i.e., adding building blocks to the PrP^Sc^ aggregate. This process is most efficient when the primary structures (amino acid sequence) of the interacting PrP molecules are identical [43]. Even a single amino acid difference can impose a significant energy barrier on the misfolding process, thus slowing or even blocking the molecular event that drives prion disease pathogenesis and transmission dynamics [44]. This explains, for a large part, the sometimes-potent genetic modulation of prion disease susceptibility observed in scrapie [45,46,47,48] and CWD [49,50,51,52], which is governed by alteration of the *PRNP* gene causing amino acid substitutions in the PrP^C^ structure.

Conversion of PrP^C^ to PrP^Sc^ was demonstrated in cell-free, in vitro assays almost 30 years ago [53]. Today, ultrasensitive methods are available for detection of PrP amyloid seeding activity, which correlates strongly with prion infectivity [54,55,56,57]. The barrier to transmission of prion disease between different species has been demonstrated in many experimental studies and has also been observed in practical husbandry.

For instance, classical scrapie in sheep has been a problem in European sheep production for about 250 years [58]. Scrapie-infected sheep were often co-housed with other production animals, horses, and pets. Still, spillover to these species was never recorded, except for goats, which are susceptible [59,60]. Human exposure must have been common, since scrapie was widely distributed, and no tests were available to remove infected animals from consumption. In most regions, wildlife, such as red deer (*Cervus elaphus*), roe deer (*Capreolus capreolus*) and other cervids, were probably also exposed by sharing grassland with scrapie-infected sheep over the centuries. It thus seems likely that a spillover of scrapie to cervids, resulting in CWD or a CWD-like disease (i.e., with subsequent horizontal transmission), would have resulted in disease outbreaks that would not have gone unnoticed. However, no such outbreaks have been recorded among European cervids, indicating a barrier for transmission of prions between sheep and cervids.

In addition, transmission properties of prions can be modulated by structural arrangements of the prion particle, implying that a PrP molecule with a given primary structure can build up PrP^Sc^ aggregates with distinct features, such as altered transmission properties [61,62].

Another important aspect of prion biology is that the above-mentioned model for prion propagation may allow a spectrum of conformational states to be propagated in parallel. This, “cloud of conformations” model, is one way of understanding prion adaptability and plasticity [63]. Different prion structures in an isolate may compete in a structure-selection process, i.e., those that most effectively misfold the available PrP substrate will dominate. This may therefore vary between host tissues and between individuals and/or species encoding different PrPs. In this way, the transmission of a prion to a new host species may elicit adaptations that alter the characteristics of the original prion structure and thereby also its characteristics, for instance concerning clinical symptoms (or lack thereof), prion tissue distribution, and transmission capacity to other species [64,65,66,67].

Thus, a prion that appears harmless to humans in its original host may, via one or more intermediate hosts, be altered so that its zoonotic potential is increased. Such alterations in transmission properties and hence zoonotic potential of prion agents are difficult to predict. Thus, the occurrence of prion diseases in humans and animals must be closely monitored and measures that minimize the entry of prions into the human food web should be continued.

## 2. Chronic Wasting Disease

### 2.1. Historical Background North America

During 1967–1979, a syndrome called chronic wasting disease was observed in 53 mule deer (*Odocoileus hemionus hemionus*) and in one black-tailed deer (*Odocoileus hemionus columbianus*) in captivity in Colorado, USA. The clinical signs appeared in adult animals and consisted of altered behavior, progressive weight loss and death within two weeks to eight months after onset of clinical signs. Diseased animals had specific CNS pathology suggesting a spontaneously occurring form of transmissible spongiform encephalopathy (TSE), not previously reported in deer species, and with an unknown origin [68].

### 2.2. Geographical Expansion, Increasing Exposure and Prevalence

A typical feature of CWD is that infected animals shed prions via saliva, feces, urine and blood, and possibly also through nasal secretions, milk and semen, and oral exposure is regarded as the main route of natural infection [69,70,71]. Susceptible hosts may be exposed to CWD prions through physical contact with an infected animal, or indirectly via contaminated food, water, and other environmental factors. In contrast to many infectious diseases in wildlife, field and modeling data from North America have indicated that CWD epizootics develop relatively slowly and that the disease remains at a low prevalence and spatially localized for a decade or more after introduction [72]. Depending on management strategy and test regimes, this may explain why the disease is often identified 10–20 years after its introduction to a cervid population [73]. However, prevalence is increasing with time after disease introduction, presumingly due to indirect transmission through contaminated food, water and the environment [74].

After the recognition of CWD in free-ranging mule deer and wapiti in 1981, a contiguous area in north-eastern Colorado, south-eastern Wyoming and western Nebraska was regarded as an enzootic region, in which CWD probably had been present for several decades prior to its recognition [72]. The introduction of CWD to Toronto Zoo probably took place via the import of infected animals from Denver Zoo, USA, and further spread of the disease from Toronto Zoo remains a possibility, but no evidence for such spread could be documented in a retrospective investigation of available material [75]. CWD was also imported to South Korea via infected live cervids [76]. More recently, CWD has been diagnosed in captive and free-ranging moose (*A. a. shirasi*) in the USA [77,78]. Since 2000, CWD has continued to spread and has been detected in many other foci in Northern America. The disease now affects 30 states in the USA and four Canadian provinces, for a detailed overview of CWD occurrence in North American wild and captive deer see [79].

### 2.3. CWD in Northern Europe

In North America, CWD has been observed in several deer species [80], including a recent case in captive reindeer (Chronic Wasting Disease Alliance, 2018), but hitherto not in free-ranging reindeer or caribou (*Rangifer tarandus*), despite overlapping habitats with other cervid species known to be affected with CWD. Inoculation studies, however, have indicated that two of three reindeer that were orally inoculated with brain homogenates from white-tailed deer (WTD) with CWD were susceptible, developing clinical signs 17–18 months post inoculation (p.i.) and died within weeks of developing clinical signs. In contrast, three reindeer inoculated in the same manner with brain homogenates from elk did not develop clinical signs and were euthanized 22–61 months p.i. [81]. Although the results could indicate that reindeer are less susceptible to elk derived CWD, the authors argue that host *PRNP* genetics are the most likely explanation. The reindeer inoculated with the elk isolate were heterozygous at codon 138 (S/N) whereas the two clinically affected reindeer inoculated with the WTD isolate were homozygous 138SS. The one that remained healthy after inoculation with the WTD isolate carried the 138S/N genotype, suggesting that this polymorphism may be protective [81]. The 138S/N polymorphism appears to be absent among Norwegian wild and semi-domesticated reindeer [49,82].

Norway hosts about 25,000 wild reindeer, distributed between 24 more or less separated populations. In March 2016, a wild European tundra reindeer (*R. t. tarandus*) was found moribund during a research field study in Nordfjella, Norway, when a reindeer flock was approached by helicopter. The animal died and was necropsied. Except for muscle hemorrhages, no other gross pathological findings were observed, but analysis of brain tissue indicated CWD [83]. This represented the first naturally occurring CWD case outside North America and the first case in a *Rangifer* subspecies. During a stamping out procedure of the Nordfjella reindeer population, 19 animals tested positive for CWD. As a result of increased surveillance of other wild reindeer populations, two cases have been diagnosed, both in the Hardangervidda population. Hardangervidda is the largest national park in Norway, hosting the largest remaining wild reindeer population in Western Europe, about 6000 to 9000 animals.

In addition to the wild reindeer, Norway hosts (2020) about 215,000 semi-domesticated reindeer of the same sub-species, the Eurasian tundra reindeer [84]. Most of the semi-domesticated reindeer in Norway is comprising a traditional cornerstone of the Sami people and culture in Fennoscandia, whereas a non-Sami reindeer herding is conducted north of the Nordfjella mountain region where CWD was first recognized. Although an exchange of animals between the wild reindeer in Nordfjella and the adjacent semi-domesticated reindeer has been observed, in particular bulls drifting north during the rut season, no CWD-positive animals have been found in this or other herds of semi-domesticated reindeer in Norway (about 57,000 animals tested, 2016–Jan. 2023). Semi-domesticated reindeer are tagged by each owner and are typically gathered twice a year, for transition to the calving ground and summer pasture regions in early spring, and again during late summer and fall for other purposes, such as tagging calves, separation of herds, selecting animals for slaughter, and parasite treatment. During the gathering and handling, reindeer are in close contact with their owners and family members, comprising the herding unit, the siida. Animals for slaughter are driven by foot if feasible, or more commonly transported on trucks to the slaughterhouse. The reindeer are subjected to veterinary inspection before and after slaughter (i.e., ante mortem control and meat control). For 2020, 52,642 reindeer were slaughtered, comprising 1,253 tons of meat, representing an economical value of about 100 million NOK [84].

### 2.4. CWD with Unusual Features in Moose and Red Deer in Northern Europe

In May 2016, two moose (*Alces alces*) were diagnosed with CWD in Selbu, not far from Trondheim, and approximately 300 km north of Nordfjella where the first reindeer case was located. Following increased surveillance of cervid populations and species in Norway, CWD has been diagnosed in 11 moose in Norway, four in Sweden [85] and three in Finland, in addition to three red deer (*Cervus elaphus*) in Norway (November 2022). Data from the investigations of moose and red deer showed that, whereas reindeer with CWD were 2.5–8 years old, CWD affected moose and red deer were 12–15 years old. In reindeer, all CWD cases tested positive for PrP^Sc^ in lymphoid tissues, whereas in moose and red deer, PrP^Sc^ deposits appeared to be confined to the CNS, and lymphoid tissues were negative [29,30,85]. Further investigations have confirmed that North American CWD strains differ from those observed in Europe, and that the European strains causing CWD in reindeer, moose and red deer are all separate strains [79,86].

The CWD cases in moose and red deer were strikingly different from CWD as observed in North America and from the outbreak in wild reindeer; in terms of age-category, organ distribution of PrP^Sc^, histopathology and epidemiology, with a seemingly sporadic appearance. By analogy to the well-established dichotomy of “classical” vs. “atypical” scrapie and BSE [87], scientists and governmental bodies in Northern Europe have arbitrarily adopted the term “atypical” CWD to distinguish the newly discovered variants in European moose and red deer, from the well-described contagious forms of CWD, reviewed in [88]. In Table 2, we use the descriptive epidemiological terms “moose sporadic CWD” and “red deer sporadic CWD”.

The expansion of CWD in North America and its appearance in Northern Europe will inevitably increase human exposure. Further, CWD prions are more diverse and adaptable than previously recognized. This diversity and adaptability are seen in both North America and Europe [86,89,90,91,92,93,94,95], suggesting that inter-species transmission properties and zoonotic potential may also be altered. The emerging dynamic character identifies CWD as a worrisome animal prion disease deserving our close attention.

In the following paragraphs we will recapitulate epidemiological, in vitro, and bioassay data addressing the zoonotic potential of CWD.

## 3. Zoonotic Potential

### 3.1. Case Reports, Epidemiological Observations, and Active Surveillance

Prion diseases have long incubation periods; in humans reaching up to fifty years [19]. The long time from potential exposure to disease manifestation makes epidemiological investigation of the zoonotic potential of animal prion disease difficult and retrospective. In addition, disease phenotypes may deviate. Although recognized as a problem, phenotypic diversity played an important role when establishing an association between variant Creutzfeldt-Jakob disease (vCJD) and exposure to BSE infected meat. The vCJD cases were unusually young (mean age around 30) as opposed to sporadic Creutzfeldt-Jakob disease (sCJD), which has a mean age of onset around 60. The clinical symptoms and disease duration also differed, and based on analyses of the *PRNP* gene, genetic prion disease could be ruled out, rendering the newly discovered disease a “new variant” of CJD [18]. The epidemic of vCJD peaked in 2000, affecting mainly UK citizens, but also appearing in many other countries [96]. Molecular analysis of proteinase K resistant PrP fragments from vCJD cases revealed a band pattern identical to that seen in cattle and rodents inoculated with material from BSE infected cattle [97]. Cases also presented with unique neuropathological features, most notably the presence of multiple kuru-like plaques, surrounded by vacuolization, clearly distinguishing the condition from sCJD [18]. In addition, the vCJD cases appeared in geographical areas that had been heavily affected with BSE 10 years earlier.

What would have been the situation if vCJD had presented disease characteristics similar or indistinguishable to sCJD; would it still have been recognized as a distinct disease and linked to BSE? The answer is “probably not”, illustrating the importance of diagnostic accuracy i.e., the ability to discriminate between similar disease pathologies and varieties of prion agents. This has been explored for sCJD [14,98,99,100], genetic Creutzfeldt-Jacob disease (gCJD) [16] and some animal prion diseases [101,102,103] and has resulted in a growing catalogue of disease sub-types and agent varieties. Thus, criteria for detailed active surveillance and diagnostics are to some extent available. Implementation of these tools in routine diagnostics and surveillance is however technically demanding and costly.

For an extensive review of the global incidence of CJD and inherent challenges related to diagnosis and surveillance see [104].

In 2006, Mawhinney and collaborators investigated the relative risk of contracting CJD for residents in CWD-endemic areas in Colorado with those living in non-CWD endemic areas [105]. The assumption was that people living in CWD-endemic areas were more exposed to CWD since most of the venison was consumed locally. They investigated a total of 65 CJD cases from 1979 through 2001 (of 506,335 deaths) and found no significant difference in CJD occurrence between the groups. Nor did they observe any increase in CJD rate in CWD affected areas, or in Colorado as a whole, concluding that CWD related human prion disease must be rare or nonexistent in Colorado.

The scientific literature contains a few case reports of rapid neurodegenerative disease in subjects with known exposure to CWD. Some of the cases have presented with unusual clinicopathological features, such as young age, but detailed analysis has failed to associate any of the cases to CWD [106,107]. Further, a cohort analysis (six years follow up) of 81 individuals attending a barbeque where CWD infected venison was unknowingly served, did not observe any neurodegenerative disease that could be linked to the exposure [108]. In conclusion, there is currently no epidemiological evidence of human prion disease caused by CWD. The datasets are however limited, for instance concerning the clinicopathological spectrum of potential human conditions caused by CWD, and the time of observations, which needs to span many decades.

### 3.2. In Vitro Amplification Methods for Assessment of Transmission Barriers

Conformational conversion of PrP^C^, seeded by the presence of preexisting PrP^Sc^ molecules, was demonstrated in cell-free in vitro systems, using purified components already in 1994 [53] and soon the barrier to transmission of prions between species was elegantly explored and demonstrated by this method [109]. In the protocol, PrP^C^ and PrP^Sc^ were mixed under denaturing conditions, with an excess of PrP^Sc^ roughly 50-fold over PrP^C^. Prior to incubation at 37 °C for two days, samples were sonicated [109]. Soto and collaborators developed this further by using fresh brain homogenates as a PrP^C^ source and by including repeated short bursts of intense sonication during the incubation, which dramatically sped up the conversion process [57]. The new method, designated protein misfolding cyclic amplification (PMCA) was highly sensitive and mimicked in vivo prion propagation, with de novo generation of infective prions, inter-species transmission potential and prion strain features [56]. This method has been used to detect and quantify prions in bodily fluids of infected animals with extreme sensitivity [110].

As an alternative to sonication, mechanical disruption of PrP^Sc^ aggregates can successfully be achieved by vigorous shaking, so-called quaking, used in quaking-induced conversion (QuIC) assays [111], which use recombinant PrP (recPrP) as substrate for the conversion reaction. QuIC assays were shown to have a sensitivity matching that of mouse bioassays (see below) [112]. Both PMCA and QuIC assays depend on handling of individual test-tubes for analysis of reaction products with western blot (WB) and are therefore less suited for high-throughput screening.

Another method known as amyloid seeding assay (ASA) also involved shaking and recPrP, but with the addition of Thioflavin T (ThT) that intercalates with misfolded PrP, and allows high-throughput multi-well readouts of fluorescence [113]. A modified, real-time version of the QuIC assay (RT-QuIC), using ThT as with ASA, but less prone to false positive signals, has been developed [114] and is today the most widely used method for ultrasensitive detection of PrP seeding activity, together with, and/or combined with the original PMCA method.

RT-QuIC and PMCA have been used to detect trace levels of amyloid seeding activity in tissues and body fluids of deer with pre-clinical or clinical CWD, such as saliva [115,116,117], urine [118], feces [119,120] and blood [121]. For a detailed comparative analysis of CWD prion detection by conventional, bioassay and amplification methods see [122]. The main advantage of the RT-QuIC method is that a standardized “universal” recombinant PrP substrate, for instance recombinant bank vole (*Myodes glareolus*) PrP, can be used to test amyloid seeding activity in tissues from a variety of different species, which makes the method well-suited for screening purposes [123]. It is also a benefit that the generated product contains no prion infectivity, which constitutes a laboratory health and safety issue. Conversely, the product generated with PMCA is infectious and the reaction depends on species and sequence specific PrP^C^ brain homogenate as substrate, which matches the incoming prion seed. This makes the PMCA method less suited for screening of samples of unknown origin but more feasible for the analysis of prion strain features and for estimating within- and inter-species transmission potential of prions [69].

Early in vitro evidence of a strong molecular barrier for transmission of CWD to humans came from a study using cell-free conversion. It was demonstrated that CWD isolates from elk, white-tailed deer and mule deer could convert human and bovine PrP, but were more than 10-fold less efficient than cervid PrP substrates, while conversion of sheep PrP was intermediate [124]. Furthermore, PMCA experiments with brain homogenates from Tg1536 mice overexpressing human PrP (MM129 genotype), gave no conversion when seeded with mule deer CWD or material from infected Tg1536 mice. Conversion of human PrP required several rounds of strain adaptation in PMCA or serial passage in transgenic mice [125], demonstrating that in vitro or in vivo adaptation of a prion strain can alter its transmission properties independent of the PrP primary structure.

To identify structural differences between human and deer PrP that impede conversion and cross-species transmission, Kurt and co-workers [126] cloned and expressed chimeric human and elk PrP, in which specific amino acids in the human PrP were substituted with those of the elk structure. They used cell lysates of transfected cells as substrate for PMCA. They did not observe any conversion of huPrP but achieved very efficient conversion with some of the hu-elk chimeric PrP substrates, results which fitted well with inoculation experiments of Tg-Hu mice and Tg-Hu-Elk chimeric mice (see below).

Further experiments with PMCA [127] have showed that CWD isolates from WTD, elk and reindeer experimentally inoculated with WTD isolate were capable of converting huPrP substrates covering the 129MM, MV and VV genotypes, although with varying efficiency. Recently, CWD isolates from six cervid species; WTD, mule deer, and elk from North America, and reindeer, red deer, and moose from Norway, were compared with the PMCA method for their inter-species transmission potential [79]. Some conversion of huPrP 129M and 129V was observed with North American CWD isolates, but no conversion was observed with any of the Norwegian isolates, suggesting that the Norwegian isolates might have a somewhat lower zoonotic potential. Conversely, the Norwegian reindeer isolate effectively converted sheep, bovine and hamster PrP, thus displaying a potential capacity to cross species barriers, comparable to that of CWD isolates from WTD. Interestingly, the Norwegian reindeer isolate had previously been shown to transmit poorly to bank vole, compared with North American CWD isolates [86].

### 3.3. Transmission of CWD to Transgenic Mice Expressing Human PrP

Natural occurrence of CWD has been recorded in several cervid species including white-tailed deer, mule deer, Rocky Mountain elk, moose, and reindeer. In addition, CWD has been experimentally transmitted to laboratory rodents and either intracerebrally and/or orally to sheep [128], cattle [129], pigs [130], cats [131], ferrets [132] and to squirrel monkeys [133]. Although this species spectrum may indicate a cause for concern, transmission of CWD between cervids is facilitated by cervid specific structural features of the prion protein [134,135,136], lowering the transmission barrier. Thus, transmission of CWD to non-cervid species, has been relatively inefficient, for instance compared with BSE.

Transgenic mice, engineered to express human PrP (huPrP, “humanized mice”) have been used to assess the human barrier for transmission of CWD (Table 3). To optimize transmission success, mouse lines that overexpress huPrP are often used. Moreover, mouse lines known to be sensitive to human prion isolates or the zoonotic BSE agent are used and infectivity of CWD isolates is demonstrated by inoculation in mice expressing cervid PrP (“cervidized” mice) or bank voles. In an elegant study, mice were engineered to express a human-elk chimeric PrP, in which four amino acids were substituted in huPrP, creating a loop sequence (aa165–177) identical to the elk PrP sequence. In contrast to huPrP mice, the chimeric (huPrP^elk165−177^) mice proved susceptible to CWD isolates, but they were concurrently less sensitive toward human CJD prions than their huPrP counterparts [134]. This study pinpointed important structural elements contributing to the barrier for CWD transmission to humans.

In prion bioassays, the primary clinical readout is progressive neurological disease. The prion disease diagnosis is then according to conventional methods confirmed by brain pathology and immunohistochemistry (IHC) detection of PrP^Sc^, often combined with WB analysis.

A challenge inherent to mouse bioassays is the short lifespan of mice (around 2.5 years) compared with the extended incubation periods frequently seen in primary transmissions of prion isolates. When primary diagnostic results are inconclusive and/or negative, other, more sensitive methods are available to test for subclinical transmission and/or asymptomatic carrier status. This is important not only to detect minute levels of PrP^Sc^, but also because prion infectivity titers do not always correlate with conventional diagnostic markers i.e., prion replication can occur without recognizable pathology and without proteinase resistant PrP^Sc^ accumulations [144]. As evident from Table 3, only two of the CWD transmission studies using huPrP mice have reported data with the aforementioned highly sensitive in vitro conversion methods or from serial passage experiments.

Race and co-workers [141] found that Tg66 and TgRM mice, overexpressing huPrP 8–16-fold and four-fold, respectively, did not develop typical or terminal prion disease after more than 700 days post inoculation with three different CWD isolates. They did not observe PrP^Sc^ deposits in IHC or PrP^Res^ fragments in WB, hallmarks of prion disease. They did, however, observe 18 clinically suspect mice of the 108 inoculated. All mice were analyzed with RT-QuIC for detection of PrP amyloid seeding activity. In four mice from the Tg66 group, results were inconclusive, reaching slightly above detection limit of the assay, suggesting that the observed clinical abnormalities could be early signs of prion disease. Race and co-workers discuss whether the RT-QuIC data could be false positive caused by residual inoculum or by the abnormally high PrP expression levels in the Tg66 mouse line, potentially causing spontaneous PrP amyloids/aggregates, detectable with the RT-QuIC method. The low number of uninoculated control mice tested was insufficient to rule out the latter possibility.

Another method for increasing prion detection sensitivity is by precipitating misfolded PrP with sodium phosphotungstic acid (PTA) prior to analysis by WB. PTA-enhancement has been shown to increase detection sensitivity for CWD approximately 100-fold compared with crude extracts [145]. In experiments with CWD inoculated huPrP mice, PTA-enhancement has not resulted in PrP^Res^ detection.

In a recently published report, Tg650 mice, overexpressing huPrP (129MM) approximately six-fold, developed unusual clinical signs with progressive myoclonus (involuntary twitching of a muscle or group of muscles) after inoculation with two CWD isolates (Wisc-1, 116AG) from white-tailed deer [143]. Despite alarming neurological signs in many inoculated mice, histopathological analysis of the brain did not indicate TSE-pathology, whereas IHC analysis was performed in six animals, of which one (#328), displayed pericellular, granular PrP deposits, in the brain. Western blot analysis of brain material from this animal was negative for PrP^Res^. Only one of the nine mice analyzed with WB was weakly positive, with an unusual two-band PrP^Res^ profile at 12 kDa and 7–8 kDa. Brain material from all mice was analyzed with a modified RT-QuIC protocol with enhanced sensitivity. With this protocol, all mice inoculated with the 116AG isolate were negative. The apparent disconnection between clinical signs and highly sensitive prion diagnostic markers suggests that the clinical signs could stem from a hard-to-detect prion agent. Unfortunately, secondary transmissions, which would provide a test for prion infectivity in these mice, were not reported. Among the Wisc-1 inoculated mice, a majority tested positive with RT-QuIC, although results also showed some inconsistencies, which was attributed to very low seeding activity. One such case was mouse #327 which had terminal illness but very low/inconsistent seeding activity in the brain. Interestingly, this mouse showed high seeding activity in feces, which was also detectable in 50% of the inoculated mice, suggesting that prion infectivity could be shed from some of the inoculated animals.

Transmission of sonicated fecal homogenate from mouse #327 to Tg650 mice and bank vole produced different results. In clinically ill Tg650 mice, no PrP^Res^ could be detected in brain homogenates and RT-QuIC analysis of the animals was not reported. In bank voles, six out of nine developed clinical symptoms. Three voles were tested for RT-QuIC seeding activity in brain and they were all positive and one (#3430) was also positive in spinal cord. Western blot analysis of brain and spinal cord homogenates from this animal revealed a typical three banding PrP^Res^ profile, dramatically different from that observed in the Wisc-1 inoculated Tg650 mice. Interestingly, the PrP^Res^ profile in the bank vole #3430 resembled that of the original WTD Wisc-1 isolate, but not the PrP^Res^ signature seen in bank voles inoculated with the WTD Wisc-1 isolate (first or second transmission).

This study [143] stands out in several ways from other investigations of CWD in humanized mice. Most strikingly, the high incidence of profound, albeit unusual, clinical signs among inoculated mice. Next, the lack of coherence between clinical signs and conventional and ultrasensitive diagnostic markers of prion disease, suggestive of toxicity driven by an easily misdiagnosed “stealth prion” evading most diagnostic modalities. The observation of seeding activity and prion infectivity in feces is also remarkable. Whether this is a phenomenon specific to the Tg650 mouse line or CWD strain, or a more widespread and previously overlooked feature of huPrP mice inoculated with CWD prions must be investigated. If the latter is shown to be the case, it will impact our understanding of the zoonotic potential of CWD, as interpreted from mouse bioassays.

Still, it can be argued from an epidemiological perspective that the traditional readout from primary prion bioassays, namely clinical neurological signs, and diagnosis of bona fide prion disease by conventional methods, provides the most relevant and informative analysis of the cross-species transmission potential for a prion. It is evident from Table 3, that primary transmission of a variety of CWD isolates to several huPrP mouse lines, overexpressing huPrP, has been uniformly negative. Although sub-passage and further use of ultrasensitive diagnostic tools, involving extra neural tissues, may identify aspects that can have been missed in earlier studies, the overall conclusion from mouse bioassays is that the human barrier for CWD transmission is very strong.

Finally, as seen in Table 3 only one of the published reports has used prion isolates from Europe [142]. Material from one reindeer and two moose CWD cases, all from Norway, were inoculated in huPrP Tg35 and Tg152c mice, covering 129 genotypes MM and VV. All inoculated mice remained healthy, and no signs of prion disease could be detected, suggesting that the human transmission barrier for these novel CWD strains is robust.

A potential weakness of the traditional Tg mouse lines, overexpressing huPrP, is that these do not precisely recapitulate tissue and organ-specific expression profiles of *Prnp* [146]. Many of the models have relatively low expression of *Prnp* in peripheral tissues, which may be important for studies of inter-species transmission potential, lymphotrophism and pathogenesis of experimental prion disease, arguing that further refinement of mouse models, for instance with gene-targeting could be beneficial [146].

### 3.4. Transmission of CWD to Non-Human Primates

The history of using nonhuman primates as models for human prion disease and in risk assessments has recently been comprehensively reviewed [147] and will therefore not be recapitulated in detail here. It is well established that the Squirrel monkey (*Saimiri sciureus*) is susceptible to both oral and intracerebral inoculation with different CWD isolates [133,148,149]. Indeed, the squirrel monkey is a permissive host to many prion agents such as kuru, vCJD, sCJD, Gerstmann-Sträussler-Scheinker disease (GSS), BSE, transmissible mink encephalopathy (TME) and sheep scrapie with relatively short incubation periods from 20 months to 46 months after intracerebral inoculation [150,151,152]. In contrast to the efficient transmission in squirrel monkeys, transmission experiments with cynomolgus macaques (*Macaca fascicularis*) have shown these to be less susceptible to animal prions, including CWD [153]. Macaques are evolutionary closer to humans [154] and considered a more precise animal model for human prion disease, although Macaque and Squirrel monkey PrPs are equally distant from human PrP [155].

Macaques have been shown to be susceptible to intracerebral inoculation of vCJD, atypical L-type BSE (L-BSE) and classical BSE (C-BSE) with incubation periods of two to three years and to sCJD with incubation period of around five years [156,157,158]. Classical scrapie was evident in a macaque after a 10-year incubation period, following a high-dose intracerebral inoculation of a classical scrapie isolate [159], illustrating the importance of very long and costly observation periods in this type of study.

In 2018, Race and co-workers [153], summarized a large study with oral and intracerebral inoculation of macaques with CWD prions. Some animals had been observed for up to 13 years after inoculation, without evidence of prion disease. The RT-QuIC assay was used to test brain, brain stem and spinal cord tissue for amyloid seeding activity, but results were similar between CWD inoculated and uninoculated animals. They observed some irregularities in the brain PrP-staining pattern of both inoculated and uninoculated animals and in two of the inoculated macaques PrP deposits that could potentially be disease-associated were observed. However, no histopathological or WB evidence of prion disease could be detected in these animals and tests with RT-QuIC were negative. Thus, the authors found no evidence of transmission of CWD to macaque.

In another, ongoing and unpublished study of CWD transmission to macaque that included oral infection with muscle tissue from cervids, preliminary congress interim reports and presentations have suggested that CWD has been transmitted to some macaques, albeit with atypical and subclinical disease manifestations. In tissues from some animals, a low level of PrP converting activity was observed with RT-QuIC and PMCA assays and sub-passage in mice overexpressing elk PrP (TgElk) or deer PrP (Tg1536) gave low attack rates, but subsequent passage from 2^nd^ passage in mice, to bank voles resulted in 100% attack rates and appearance of typical prion disease pathology. Interestingly, infectivity was found also in the gastrointestinal tract [160]. These findings indicate that the species barrier to humans is not absolute, and it is likely that it can be crossed (Schätzl, personal communication).

Full comparative analysis of the two apparently contradicting macaque investigations must await publication of the latter, still ongoing investigation. However, both studies clearly demonstrate that the barrier for transmission of CWD to macaque is very strong, but probably not absolute, which is in accordance with data from transgenic mice and in vitro experiments. Differences between studies could be related to differences in CWD inocula i.e., strain differences and infective doses as well as differences among the recipient macaques.

## 4. Discussion

We have summarized available epidemiological, in vitro and bioassay derived data concerning the zoonotic potential of CWD. We have identified only one report in which CWD strains recently identified in Northern Europe have been analyzed for their zoonotic potential, by inoculation in huPrP mice [142] and one study exploring this by in vitro methods [79]. Since CWD strains identified in Northern Europe clearly are different from strains from North America, further experiments are needed (and ongoing) to map this out in further detail.

Data from recent bioassays in mice and macaques suggest that conventional readouts for prion disease should be strengthened by ultra-sensitive RT-QuIC and PMCA assays in combination with serial passage to analyze for prion infectivity. The phenomenon of unusual/atypical clinicopathological disease presentation and proteinase sensitive prion strains, evading traditional PrP^Sc^/PrP^Res^ detection, is still incompletely understood, including its real-life epidemiological relevance. For instance, are the rare observations of abnormal PrP deposits in peripheral tissues in healthy individuals merely rarities reflecting the ultrasensitive methods used, or representations of phenomena directly relevant to surveillance programs and risk assessments? We know that prion agents can adapt and change characteristics when propagated within the host or in a new host according to mechanisms that are poorly understood.

Controlling a transmissible and potentially zoonotic disease in wild cervid populations is complicated, and many disease characteristics, such as long incubation time, no antibody production (i.e., no immunity), pathogen robustness in the environment and many other factors are further challenges to surveillance strategies. Furthermore, most of the affected cervid populations are in remote areas with restricted availability and infrastructure. Today, the management of these cervid populations in Fennoscandia is based on hunting, with a private motivation for preparing and consuming the game. During the culling of the affected reindeer population in Nordfjella, where CWD was first diagnosed, hunted carcasses were held in arrest until CWD test results were available. This practice, however, is very time consuming and costly, and may be evaluated against the precautionary principles. Thus, the appearance of CWD in wild cervids in Fennoscandia necessitates new management practices, for Norway and for the European Union.

A major goal for the management of CWD in Norway has been to prevent the disease from entering the semi-domesticated reindeer herds [161]. The non-Sami reindeer herding is conducted north of and in close proximity to Nordfjella, and exchange of animals between wild and semi-domesticated herds have been observed, opening for the possibility that infected wild reindeer may already have had contact with reindeer herding. However, about 14,000 semi-domesticated reindeer from these herds (Jan. 2023) have been tested with no CWD-positive animals detected [162].

It is important to keep in mind that also semi-domesticated reindeer are free-ranging year around just as much as the wild reindeer, and only gathered and handled a couple of times during the year. Despite being routinely inspected and herded, it is challenging to address disease among free-ranging animals in remote high mountain pastures, and fallen stock is quickly scavenged and decomposed making it difficult to address cause of death.

Exposure of people through consumption is very similar whether it is a wild, hunted reindeer or a semi-domesticated, slaughtered reindeer. Reindeer herders are probably consuming more reindeer meat than the general consumer. In addition, their work involves close contact with reindeer during gathering and handling of animals, but also through periods of supplementary feeding which is becoming increasingly common. Although the chance of CWD eradication may be greater in a semi-domesticated reindeer herd than in the wild populations, an appearance of CWD in reindeer herding will necessitate dramatic measures which may have a major impact on the herd size and structure, the use of pastures, collaboration between herders, the economy, as well as the social, traditional, and cultural aspects associated with reindeer herding.

## 5. Conclusions

No cases of human prion disease caused by CWD have been reported and most experimental data suggest that the zoonotic potential of CWD is very low. Based on the current knowledge and identified knowledge gaps regarding the zoonotic potential of the new CWD strains in Fennoscandia, it is good advice to keep human and animal exposure to prions to an absolute minimum and closely monitor and restrict CWD and other animal prion diseases to prevent these agents from entering the human food chain.

## Figures and Tables

**Table 1:** Human prion diseases and their epidemiological profile.

Disease	Mode of Occurrence	References
Creutzfeldt-Jakob disease		
Sporadic, sCJD	Sporadic	[14]
Sporadic fatal insomnia	Sporadic	[15]
Genetic CJD, gCJD	Familial, *PRNP* mutations	[16]
Iatrogenic CJD, iCJD	Acquired, medical or surgical treatment	[17]
Variant CJD, vCJD	Acquired, foodborne zoonosis	[18]
Kuru	Acquired, cannibalism (disease eradicated)	[19]
Gerstmann-Sträussler-Scheinker disease, GSS	Familial, *PRNP* mutations	[20]
Fatal familiar insomnia, FFI	Familial, *PRNP* mutations	[20]
Variable proteinase sensitive prionopathy VPSPr	Sporadic	[21]

**Table 2:** Animal prion diseases and their epidemiological profile.

Disease and Species of Occurrence	Mode of Occurrence	References
Scrapie in sheep and goats		
Classical	Contagious	[22]
Atypical/Nor98	Sporadic	[23]
Bovine spongiform encephalopathy in cattle, BSE		
Classical C-BSE	Foodborne	[24]
Atypical L-BSE	Sporadic	[25,26]
Atypical H-BSE	Sporadic	[25,27]
Chronic wasting disease in deer, CWD		
Classical C-CWD	Contagious	[28]
Moose sporadic CWD, Mo-sCWD	Sporadic	[29]
Red deer sporadic CWD, Rd-sCWD	Sporadic	[30]
Camelid prion disease	Contagious	[4]
Transmissible mink encephalopathy TME	Foodborne (BSE L-form)	[31]
Transmissible feline encephalopathy FSE	Foodborne (C-BSE)	[32]

**Table 3:** Chronic wasting disease transmission experiments with transgenic mice expressing Human PrP (“humanized mice”).

CWD Source		huPrP, 129MV	Readouts							Reference
North America	Europe		Clinical signs	Brain pathology, IHC, PrP^Sc^	WB PrP^Res^	Other	RT-QuIC	PMCA	Serial passage	
Elk	NA	Tg40,1X,129M Tg1, 2X, 129M	Neg. (0/29) Neg. (0/22)	NA	Neg.	PTA Neg.	NA	NA	NA	[137]
Elk, MD ^1^, WTD ^2^	NA	Tg440, 2X	Neg. (0/67)	Neg. (selected mice tested)	NA	NA	NA	NA	NA	[138]
MD	NA	Tg152, 2X 129VV Tg45, 4X 129MM Tg35, 6X, 129MM	Neg. (0/41)	Neg.	Neg.	PTA Neg.	NA	NA	NA	[139]
WTD	NA	HuMM129, 1X HuMV129, 1X HuVV129, 1X	Neg. (0/72)	NA	NA	IDEXX Spleen, Neg.	NA	NA	NA	[140]
Elk and MD	NA	Tg(huPrP) 1-2X Tg(huPrP^elk166−174^)	Neg. (0/12) Pos. (7/8 Elk CWD), (3/4 MD CWD)	Neg. Pos.	Neg. Pos.	PTA Neg. Pos.	NA	NA	NA	[126]
Elk, WTD, MD	NA	Tg66, 8-16X 129M TgRM, 2-4X 129M	4/52 suspicious 0/45	Neg.	Neg.	PTA Neg.	Inconclusive	NA	NA	[141]
	One reindeer, two moose	Tg35 2X, 129VV, Tg152c 6X 129MM	0/19 RD CWD 0/39 MO CWD	Neg.	Neg.	NA	NA	NA	NA	[142]
WTD, Wisc-1, 116AG isolates	NA	Tg650, 6X, 129MM	Myoclonus, variable CNS signs in 93.8%	1/5, remaining animals NA	1/20	NA	7/18 Pos. Brain 8/18 Neg. brain 3/18 Inconclusive	NA	2nd passage to Tg650 mice 5/10 Pos. Bank vole 4/9 Pos.	[143]

^1^ Mule deer, ^2^ White-tailed deer.

## Data Availability

Not applicable.

## Abbreviations

ASA	Amyloid seeding assay is a method by which recombinant prion protein is polymerized into amyloid fibrils in the presence of partly purified prion preparations. The newly generated fibrils that can be detected with dyes like Thioflavin T.
BSE	Bovine spongiform encephalopathy is a prion disease of cattle caused by prion-contaminated meat and bone meal.
CJD	General abbreviation of Creutzfeldt-Jakob disease in humans
sCJD	Sporadic Creutzfeldt-Jakob disease, caused by spontaneous conversion of PrP^C^ into PrP^Sc^ or by somatic mutation in PRNP which is the gene encoding PrP.
sFI	Sporadic form of fatal insomnia. Extremely rare sporadic form of the inherited familial fatal insomnia
FFI	Inherited prion disease caused by germ-line mutation in PRNP.
gCJD	Inherited form of Creutzfeldt-Jakob disease, caused by germ-line mutation in PRNP.
iCJD	Creutzfeldt-Jakob disease, caused by infection with prion-contaminated tissue grafts or medical preparations.
vCJD	Variant Creutzfeldt-Jakob disease, caused by BSE-contaminated feedstuff
CM	Cynomolgus macaques, Macaca fascicularis, Old World monkey used in experimental transmission studies of prion diseases, to test for zoonotic potential
CWD	Chronic Wasting disease, is an infectious prion disease affecting cervid species
FSE	Feline spongiform encephalopathy is prion disease of fields caused by intake of BSE-contaminated feedstuff
GSS	Gerstmann-Straussler-Sheinker syndrome is a human prion disease caused by germ-line mutations in PRNP
IHC	Immunohistochemistry is a commonly used method for selective identification of proteins in biological tissues by use of antibodies that binds specifically to the proteins of interest.
Mo-sCWD	Moose sporadic CWD is a prion disease recently identified in Fennoscandia (Norway, Sweden, and Finland). The disease has an apparently sporadic occurrence, affecting old animals, and prions appear confined to the central nervous system i.e., not detectable in peripheral lymphoid tissues. Our understanding of this disease, including its epidemiology and potential to infect other species is still incomplete and an area of intense investigation.
PMCA	Protein misfolding cyclic amplification is a method whereby in vitro nucleation-dependent conversion of PrP^C^ into PrP^Sc^ is accelerated by use of periodic fragmentation of PrP^Sc^ fibrils by intense bursts of ultrasonic waves, followed by incubation, allowing new PrP^Sc^ fibrils to form, amplifying the original signal. The PMCA method is used for ultrasensitive detection of prions in tissue samples of environmental samples, and for investigation of many aspects of prions.
PRNP	The gene encoding the prion protein
PrP	General abbreviation of the prion protein
PrP^C^	The physiological cellular prion protein
PrP^Res^	A misfolded and proteinase K resistant protein core of the prion protein detected in gel-electrophoresis and protein immunoblots (western blots)
PrP^Sc^	An abnormal, pathogenic, and infectious conformer of the prion protein, isolated from patients with prion disease
recPrP	Recombinant prion protein, produced in bacteria
huPrP	Human prion protein
PTA	Phosphotungstic acid is used to precipitate and thus concentrate prions from tissue preparations to enhance detection sensitivity
QuIC	Quake induced conversion is a method for sensitive detection of misfolded PrP by in vitro conversion of an excess of recombinant PrP in the presence of a tissue derived seed, for instance from an animal suspected to be prion infected. While the PMCA method uses ultrasound to break apart PrP fibrils, the QuIC method achieves this by vigorous shaking (quaking).
Rd-sCWD	Red deer sporadic CWD is a prion disease observed in three red deer in Norway with what appears to be sporadic occurrence. Prions appear confined to the central nervous system i.e., not detectable in peripheral lymphoid tissues. Our understanding of this disease, including its epidemiology and potential to infect other species is still incomplete and an area of intense investigation.
RT-QuIC	Real-time quake induced conversion is a modified and improved variant of the QuIC method, allowing real-time detection of newly formed PrP aggregates with fluorescence detection of thioflavin T. The RT-QuIC method allows ultrasensitive detection of misfolded PrP in tissue samples, lymph, and environmental samples.
SM	Squirrel monkey, Saimiri sciureus, New World monkey, used in experimental transmission studies of prion diseases, to test for zoonotic potential.
ThT	Thioflavin T is a fluorescent dye which binds to proteins rich in beta-sheet structures, such as amyloid. Upon binding, the dye displays an enhanced fluorescence and emits at about 480 nm after excitation at 450 nm. ThT is widely used for detection of amyloid protein aggregates in tissues and in vitro, for instance with the RT-QuIC method.
TSE	Transmissible spongiform encephalopathy is a previously used term for the group of neurodegenerative diseases today known as prion diseases
WB	Western blot, a commonly used method for analysis of proteins, separated with electrophoresis and transferred to membranes for specific detection with antibodies raised against the protein(s) of interest. The term Western stems from a lab-jargon following a method for detection DNA, named after its inventor Edwin Southern. Similar detection of RNA is called Northern blot.
WTD	White-tailed deer, Odocoileus virginianus.
Zoonosis	An infectious disease that can be transmitted between animals and humans
